# Influence of repetitive diamond bur use on the trueness and surface roughness of subtractively manufactured PICN restorations

**DOI:** 10.2340/biid.v13.46232

**Published:** 2026-06-17

**Authors:** Xiaoyun Liu, Andrew B. Cameron, Ketil Hegerstrøm Haugli, Adriane Andersen Mougios, Nicholas C. K. Heng, Joanne Jung Eun Choi

**Affiliations:** a Sir John Walsh Research Institute, Faculty of Dentistry, University of Otago, Dunedin, New Zealand; b School of Medicine and Dentistry, Griffith University, Gold Coast, Australia; c Oslo Metropolitan University, Oslo, Norway

**Keywords:** CAD/CAM, subtractive manufacturing, PICN, hybrid ceramic, bur degradation, fabrication trueness, surface roughness

## Abstract

**Objectives:**

Progressive diamond bur degradation during the subtractive manufacturing (SM) of polymer-infiltrated ceramic networks (PICNs) compromises restorative clinical quality. To evaluate the degradation extent across two SM systems and investigate its effects on the trueness and surface roughness of PICN crowns.

**Materials and Methods:**

A total of 121 premolar crowns were fabricated from PICN blocks (Vita Enamic) using two SM machines (PrograMill PM7 and inLab MC X5). Crown trueness was quantified by superimposing crown scans onto the original digital design using three-dimensional inspection software (Geomagic Control X) and reported as the root-mean-square (RMS) ± standard deviation (µm). Surface roughness (Ra, Sa, Sq) was measured using confocal microscopy. Bur degradation was assessed after every five crown fabrications using machine-reported lifespan, surface roughness, and color deviation maps. Diamond bur tips, before and after use, were examined using scanning electron microscopy to characterize wear patterns. Statistical comparisons were performed both between and within machines using Welch’s Analysis of Variance (ANOVA) (*p* < 0.05).

**Results:**

Significant differences were observed in regional trueness (*p* < 0.0001): the PM7 yielded more accurate intaglio (inner) surfaces (22.21 ± 4.31 µm), while the MC X5 achieved higher external surface trueness (17.89 ± 2.76 µm). Bur degradation led to distinct error modes – whereas the PM7 exhibited a significant increase in negative deviations (overmilling), the MC X5 predominantly resulted in under-milled surfaces but with less impact from bur degradation. Crown surface roughness also differed between the two SM systems. While a consistent decreasing trend in surface roughness was observed in the MC X5 with repetitive milling cycles, the PM7 showed an initial decline followed by an accelerated increase after the 55th crown.

**Conclusions:**

The rate and extent of bur degradation varied by systems, causing distinct impacts on crown quality. Degradation in the PM7 predominantly resulted in adverse over-milling errors, whereas the MC X5 was more likely to cause inadequate material removal. System-specific degradation mechanisms substantially affect the ultimate quality of restorations. Surface roughness initially decreased in both SM systems; however, the systems followed divergent late-stage trajectories – one maintained a downward trend while the other exhibited a U-shaped recovery.

## Introduction

The optimal goal of restorative dentistry is to replace lost dental hard tissues with a restorative material that possesses biomimetic properties and aesthetic qualities that work in harmony with the oral environment [1]. Although dental ceramics and resin composites are the primary non-metallic materials, choosing a final restorative material often involves a clinical compromise between the two.

Glass-ceramics are considered more aesthetically appealing and have superior translucency that closely resembles natural tooth enamel [2]. However, their inherent brittleness and high rigidity render them more susceptible to chipping, thereby increasing the likelihood of breakage during manufacturing processes [3]. Resin composite materials provide greater flexibility and ease of machining but suffer from higher wear and inferior aesthetic stability [4]. To enhance material performance, the ‘Polymer-Infiltrated Ceramic Network (PICN)’ was developed. This hybrid material integrates a porous ceramic scaffold with an infiltrating polymer, a composition that aims to combine the distinct advantages of both materials, such as the stiffness of the ceramic and the fracture toughness of the polymer [5]. It is typically created by infiltrating polymerizable monomers into a pre-sintered ceramic network, followed by curing through heat-induced polymerization [6, 7]. Vita Enamic is a well-known PICN hybrid material in restorative dentistry, which features a sintered ceramic matrix (86 wt%) infiltrated with a polymer matrix (14 wt%) [8]. As heterogeneous materials, PICNs aim to reduce the brittleness and rigidity characteristic of ceramics while incorporating the flexibility and machinability of polymers [2].

Subtractive manufacturing (SM; also known as milling) is the dominant manufacturing method for dental ceramic restorations and is valued for its long-standing clinical success [9]. This process utilizes computerized numerical control (CNC) machines to precisely mill restorations from a solid block of material with high-speed cutting tools [10]. A key advantage of SM is that it reshapes pre-manufactured, fully-densified PICN materials that are already in their final, polymerized state [11]. This means no polymerization occurs after milling, preventing the polymerization shrinkage common in other methods (e.g. 3D printing) [12]. This absence of chemical reaction ensures high dimensional stability in the manufacturing process and reduces issues such as porosities and inhomogeneous consistency [10].

Following the digital design phase, the computer-aided manufacturing (CAM) software of the SM machine processes the computer-aided designed (CAD) model to generate toolpaths, which are the specific paths that milling burs will follow [13]. G-codes are the programming language used to operate SM machines, controlling tool movement, feed rates, spindle speeds, tool changes, and coolant management during wet grinding [14]. These commands are often referred to as milling strategies, which are predefined, material-specific protocols typically established by machine manufacturers [15]. The logic for roughing and finishing passes, tool step-over, and pathing can vary widely and are not interchangeable between different CAM systems. Thus, different milling systems are likely to produce varying final restoration quality, even when fabricating from an identical CAD model [15]. Another critical factor that introduces variability within a single SM system is the condition of the milling tool, which experiences progressive wear with each cycle [15, 16]. This wear, visible in the rounding of cutting edges or the loss of diamond grit, causes deviations from the machine’s programmed parameters and leads to the transfer of increased cutting forces and thermal stress to the restorative material, potentially affecting its integrity or surface quality [17, 18].

The dimensional fidelity of a restoration is essential for its clinical longevity. in accordance with the ISO 5725-1, manufacturing accuracy is assessed by evaluating both trueness (the dimensional deviation of the milled restoration from the original digital design) and precision (the manufacturing stability among multiple SM restorations obtained under stipulated conditions) [16]. Fabrication trueness is paramount to ensure accurate fit of restorations, a decisive factor in their long-term clinical success [17]. An ill-fitting crown can lead to marginal gaps, microleakage due to cement washout, secondary caries, plaque buildup, and issues related to retention and resistance [18]. While precision ensures the consistency and predictability of the SM process, trueness is directly threatened by the manufacturing process itself. Therefore, any uncompensated factor, such as the gradual wear of a diamond bur, can introduce systematic errors that reduce fabrication trueness and threaten the restoration’s success. The surface topography of a restoration is another crucial factor influencing mechanical strength, biocompatibility, and antagonist wear. During SM, the grinding action of high-speed diamond burs on PICN blocks inevitably produces surface flaws, such as microcracks, chipping, pits, and pores [19]. These defects serve as stress concentration points, which are the leading cause of failure in ceramic-based restorations [20, 21]. The functional strength of brittle materials is inversely related to the size, sharpness, and depth of surface flaws, which collectively determine surface roughness [22]. Increased roughness increases surface area and significantly affects initial adhesion and microbial retention, thereby promoting biofilm formation; if roughness is present at subgingival margins, microbial buildup increases further, raising the risk of secondary caries and periodontal inflammation [23, 24]. A rough surface also acts as an abrasive, accelerating wear of the opposing dentition [25].

The specific impact of bur degradation on PICNs remains largely unexplored. The unique dual-network microstructure of PICN presents distinct machinability challenges compared to other ceramics or resins. There is a critical lack of study regarding how progressive bur wear interacts with this specific material across different SM systems. This knowledge gap carries significant clinical implications as dental practitioners risk utilizing burs beyond their effective lifespan. This can result in manufactured restorations with compromised adaptation, unacceptable surface roughness and increased risks of clinical failure. Therefore, this study aimed to (1) quantify the progressive wear of diamond burs during repeated milling cycles, and (2) assess how this cumulative bur wear affects the dimensional trueness and surface roughness of subtractively manufactured PICN crowns. The null hypotheses were that repeated bur use (i.e. progressive bur wear) would have no statistically significant impact on the trueness or surface roughness of the fabricated PICN crowns, and that there would be no statistically significant differences in crown quality produced by different SM machines.

## Materials and methods

### Tooth preparation and crown design

A maxillary left second premolar (tooth 15) from a typodont (D955HP-200(GUB)-MF, Nissin Dental Products Inc., Kyoto, Japan) was prepared for a full-anatomical crown. The preparation parameter included a 1.5-mm occlusal reduction at the cusps and 1.0 mm in the central fissure, 0.8–1.0 mm of axial reduction, and a rounded shoulder finish line. After the abutment and its antagonist were scanned with a desktop scanner (3Shape E4, 3Shape A/S, Copenhagen, Denmark), the premolar crown was digitally designed in CAD software (3Shape Dental System version DS2022-1/2.22.2.0, 3Shape A/S, Copenhagen, Denmark) with a 50-μm internal cement gap and a 25-μm marginal cement gap beginning 1.0 mm from the finish line. Due to the size limitations of diamond burs used in SM, a drill compensation corresponding to the smallest bur diameter of 0.6 mm was applied to the design.

**Figure 1:**
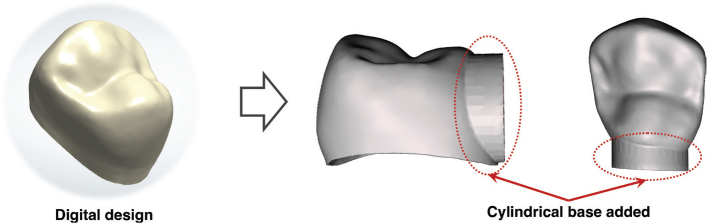
Crown modification by adding a cylindrical plate to the palatal surface for calibration between subtractive manufacturing machines.

Following the initial design, the premolar design was imported into open-source CAD software (Meshmixer, Autodesk Inc., San Rafael, CA, USA) to add a cylindrical plate to its palatal surface (Figure 1). This plate acted as a standardized reference, guaranteeing that the orientation and coordinate system of the crowns were consistently aligned across various CAM systems. The modification was inspected and verified as not interfering with any other crown areas required for analysis. The Standard Tessellation Language (STL) file of the modified crown served as the definitive reference model for all subsequent manufacturing processes and trueness analyses.

### SM process

**Figure 2:**
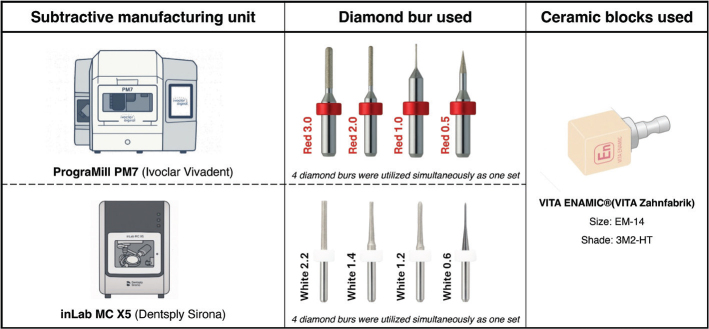
Subtractive manufacturing machines and their diamond burs used for fabricating PICN restorations.

The reference STL file was imported into two CAM software programs: PrograMill CAM (v4.13, Ivoclar Vivadent, Schaan, Liechtenstein) and inLab SW (v22, Dentsply Sirona, Bensheim, Germany). These programs generated the milling toolpaths (G-Codes) for their respective SM machines, the PrograMill PM7 and inLab MC X5 (Figure 2). Both systems operated under wet grinding conditions and coolant maintenance was performed in strict accordance with the manufacturers’ monitoring protocols. For the inLab Mac X5, a water-based lubricant mixture (Dentatec, Dentsply Sirona) was used, while the PrograMill PM7 utilized its proprietary cooling fluid (PrograMill Fluid, Ivoclar). All crowns were milled from Vita Enamic blocks (Shade: 3M2-HT) using a set of four diamond burs by each machine.

**Figure 3:**
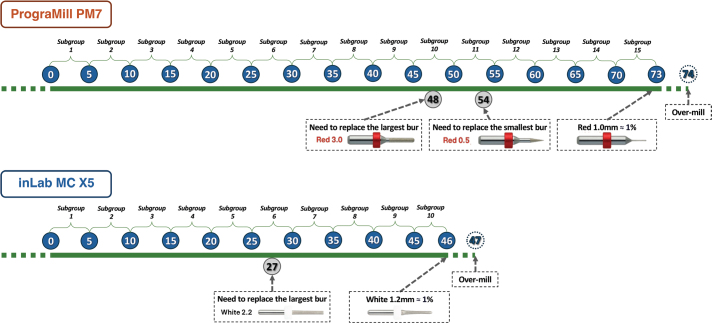
Subtractive manufacturing processes and sample size for each group.

In both machines, the largest burs (PM7: Red 3.0; MC X5: White 2.2) were used for bulk material removal, while finer burs were employed for final shaping. The experimental endpoint was designed to test the full lifecycle of the primary finishing burs; therefore, milling cycles were repeated continuously in each system until the CAM program reported the key finishing bur (PM7: Red 1.0; MC X5: White 1.2) was 100% worn (i.e. the tool life shown fell below 0%). The designated reference burs were the ‘Red 1.0’ for the PM7 and the ‘White 1.2’ for the MC X5 (Figure 3). To complete the fabrication of the final crowns and fully utilize these diamond burs, they were used for less than 2% beyond their manufacturer-designated lifespan. This resulted in different total sample sizes: the PM7 produced 74 crowns, while the MC X5 produced only 47. The sample sizes for each group were determined according to the varying bur lifespans and milling strategies associated with each system. Other burs in the set were replaced as needed according to manufacturer guidelines. In the PM7, the Red 3.0 and Red 0.5 burs were replaced after milling 48 and 54 crowns, respectively. The White 2.2 bur in the MC X5 was replaced once after 27 crowns. To assess the effect of progressive bur degradation, the subtractively-manufactured restorations were grouped into sequential subgroups of five crowns. This specific interval was established based on a preliminary pilot test that showed a 5-crown size provided optimal resolution to detect significant, progressive stages of bur wear while filtering out baseline manufacturing noise. There were 15 subgroups milled by the PM7, but only 10 by the MC X5 (Figure 3). For clarity, the milling machine and associated rotary tools are collectively referred to by the SM system. This terminology explicitly acknowledges that within the proprietary closed ecosystems of these devices, the integration of hardware and software constitutes a single, composite variable.

### Diamond bur degradation assessment

The degree of diamond bur wear was assessed and measured across a four-part protocol after milling every five crowns (per subgroup): (1) bur lifespan reported by CAM programs, (2) average surface roughness of the bur tips, (3) color deviation maps generated by 3D inspection software, and (4) observations through scanning electron microscopy (SEM).

#### CAM program recording

The software-reported wear was documented by directly recording the percentage of remaining usable lifespan for each diamond bur from the PrograMill7 and inLab CAM software. The average working time (in min) expended by each bur to fabricate a single crown was calculated.

#### Surface roughness

**Figure 4:**
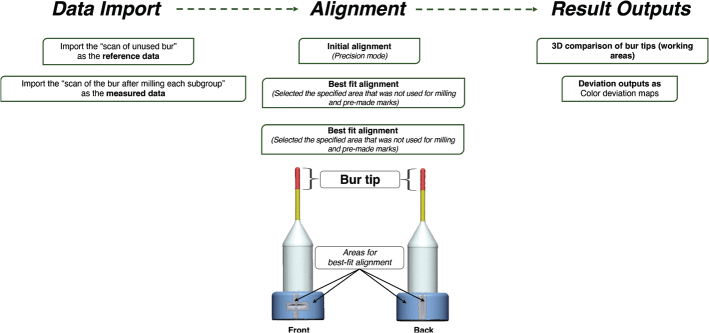
Analysis of the bur degradation before using and after each subgroup – color deviation maps generated for comparison between new and used diamond bur surfaces.

The surface roughness of diamond bur tips was measured with a LEXT OLS5100 laser scanning microscope (Olympus Corp., Tokyo, Japan). To reduce light reflection from diamond particles, a thin layer of anti-reflective powder (Helling 3D Laser Scanning Spray, Helling GmbH, Heidgraden, Germany) was applied to cover the scanned bur surfaces. To ensure standardized orientation and repeatable measurements, two marks (‘cross’ and ‘vertical line’) were cut on the bur shanks (non-working regions). These marks enabled consistent identification of the ‘front’ and ‘back’ surfaces of the bur for downstream analysis (Figure 4).

All roughness measurements were limited to the corresponding specific region of interest (ROIs) defined on the active cutting tip of the burs, as explicitly defined in Table 1. These ROIs were selected as they represent the primary functional cutting surfaces and are thus the expected sites of maximum wear. To ensure consistent re-evaluation, the precise coordinates and boundaries of each ROI were saved. This measurement template was then reloaded for all subsequent time points, guaranteeing that the identical surface area was analyzed after each milling cycle. For each bur, two separate measurements of the areal surface roughness were taken – one on the front surface and one on the back. Before calculating roughness, an ‘F-operation’ filter (form removal) was applied to the measured surface to remove the underlying macroscopic geometry of the bur (i.e. the basic cylindrical shape). The following areal surface roughness parameters were then calculated for the entire ROI: arithmetic mean height (Sa), root mean square height (Sq), skewness (Ssk), and kurtosis (Sku). The Sa and Sq indicate the average vertical height difference and standard deviation (SD), respectively, of the surface height distribution, whereas the distribution of surface texture heights is described by the Ssk and Sku parameters [26]. The profile surface roughness was displayed along five parallel measurement lines aligned with the bur shape within the ROI.

**Table 1:** The region of interest (ROI) is defined and measured for each diamond bur.

PrograMill PM7		inLab MC X5	
**Red 3.0**	1,021 × 1,021 pixels (≈ 1,280 × 1,280 µm^2^)	**White 2.2**	1,021 × 1,021 pixels (≈ 1,280 × 1,280 µm^2^)
**Red 2.0**	1,021 × 1,021 pixels (≈ 1,280 × 1,280 µm^2^)	**White 1.4**	1,021 × 840 pixels (≈ 1,280 × 1,052 µm^2^)
**Red 1.0**	1,021 × 660 pixels (≈ 1,280 × 827 µm^2^)	**White 1.2**	1,021 × 890 pixels (≈ 1,280 × 1,115 µm^2^)
**Red 0.5**	1,021 × 255 pixels (≈ 1,280 × 320 µm^2^)	**White 0.6**	1,021 × 570 pixels (≈ 1,280 × 715 µm^2^)

#### Color deviation map

To quantify geometric wear, each diamond bur was scanned using the 3Shape E4 desktop scanner and exported as an STL file both before use and after fabrication for each subgroup. All burs were coated with the previously described anti-reflective spray (Helling 3D Laser Scanning Spray) prior to scanning. The scan of the new, unused diamond bur served as the reference data. After importing the scans of the used burs into Geomagic Control X inspection software (version 2022.1, 3D Systems, Rock Hill, SC, USA), they were superimposed with their corresponding reference data using the initial and best alignment procedures. The 3D comparison analysis focused on the working regions (diamond bur tips), and the qualitative results consisted of color deviation maps, which illustrated dimensional changes across the bur surfaces. Since the spray powder layer has an average thickness of approximately 16 µm [27], a tolerance of ±20 µm was set for the color bar scale. This range effectively defined the nominal ‘no-change’ or acceptable deviation threshold for the analysis.

#### Scanning electron microscopy

For micro-morphological analysis, representative diamond burs from each CAM system were examined in two states: new (unused) and after completing the final milling cycle. Intermediate analysis during the milling process was not feasible, as the required sample preparation for SEM, especially sputter-coating, is a terminal procedure that makes the bur unusable for further milling. Before imaging, samples were mounted on aluminum stubs using double-sided carbon tape and sputter-coated with approximately 10 nm of gold/palladium (Au/Pd) using a Quorum Q150V Plus modular coating system (Quorum Technologies Limited, East Sussex, UK). The bur tips were then analyzed in a field-emission scanning electron microscope (FESEM) (Zeiss Sigma 300VP FESEM, Carl Zeiss Inc., Oberkochen, Germany) at an accelerating voltage of 5 kV. Micrographs were taken at 100x, 200x, and 300x magnification to qualitatively evaluate primary wear mechanisms, such as diamond grit dislodgement, abrasive flattening (blunting), and clogging of the metallic matrix.

### Trueness and surface roughness analysis of crowns

#### Trueness

**Figure 5:**
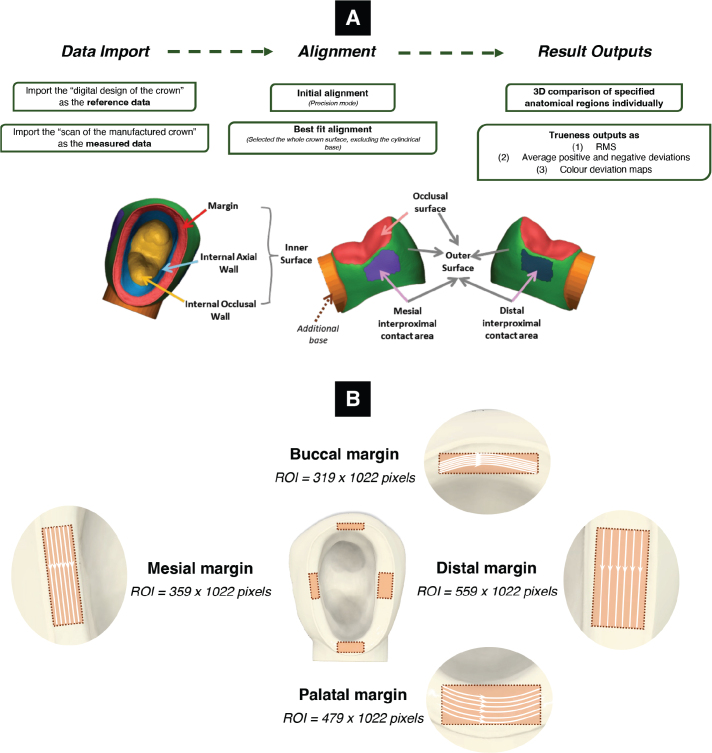
The process to measure the crown quality – (A) Trueness of various anatomical regions of the crowns in Geomagic Control X; (B) Surface roughness of crown margins. ROI: region of interest.

Following SM, each crown specimen was ultrasonically cleaned in distilled water for 5 min to remove residual coolant, air-dried and stored dry in ambient laboratory conditions until optical digitization using the 3Shape E4 scanner, which was regularly calibrated to minimize thermal drift. The intaglio (internal) and external surfaces of the crown were scanned separately. This process was repeated three times for each surface to ensure high reliability and minimize the potential impact of random optical measurement errors, and all resulting STL files were imported into the 3D inspection program (Geomagic Control X). Each crown scan was individually superimposed on the definitive reference STL (the modified crown design shown in Figure 1). Initial alignment was performed in precision mode, followed by a best-fit alignment of the entire crown surface, excluding the additional cylindrical plate (Figure 5A).

Fabrication trueness was quantified using the root mean square (RMS) value, which represents the average magnitude of deviation between the crown and its reference design, with the following formula [28]: *(where, Sample data = PICN crown from subtractive manufacturing and Reference data = Digital design of the crown)*


$$RMS=\sqrt{\frac{\sum_{i=1}^{n}{\left(x_{sample\;data,i}-x_{reference,i}\right)}^{2}}{n}}$$

This 3D comparison was performed on the entire inner and outer surfaces of crowns, as well as on several anatomical regions: margin and internal axial and occlusal walls, which formed the intaglio surface; occlusal and mesial and distal interproximal contacts belong to the outer surface. The SD of the RMS values was calculated to assess variability in trueness across multiple crowns within each subgroup. The average positive and negative deviations of the outer and inner surfaces were also calculated. For qualitative visualization, color deviation maps were generated for representative crowns from each subgroup. Based on clinical literature, the color bar range was set to the clinically acceptable threshold of ± 120 µm [29]. Within this scale, the tolerance (the ‘green’ band) was set to ± 30 µm to represent the ideal fabrication trueness [30, 31].

#### Surface roughness of crown margins

The surface roughness of fabricated crowns was quantified at the clinically critical marginal regions. Four aspects (buccal, palatal, distal and mesial) were measured on each crown using the LEXT OLS5100 laser scanning microscope. The ROI for each measurement was defined by the anatomical width of the margin, resulting in four distinct acquisition sizes. These ROIs were 319 × 1,022 pixels (≈ 400 × 1,280 µm^2^) for the buccal margin, 479 × 1,022 pixels (≈ 600 × 1,280 µm^2^) for the palatal, 559 × 1,022 pixels (≈ 700 × 1,280 µm^2^) for the distal, and 359 × 1,022 pixels (≈ 450 × 1,280 µm^2^) for the mesial margin. Due to the curved spherical geometry of the buccal margin, an ‘F-operation’ filter was applied only to this region before the calculation.

The areal surface roughness (Sa, Sq) for each crown was then calculated as the average of the four individual marginal measurements. Within each of the four marginal ROIs, six profile measurements were assessed along parallel lines (either straight or curved), along with the toolpath of diamond bur movement. The average of all 24 linear measurements (6 lines × 4 margins) was used to determine the final profile roughness values (Ra) for the crown (Figure 5B).

### Statistical analysis

All statistical analyses were conducted using Prism 10 (GraphPad Software, LLC, San Diego, CA, USA), with a significance level of *p* = 0.05. Descriptive statistics (mean and SD) were calculated for all continuous dependent variables, including dimensional trueness (RMS, average positive and negative deviations) and surface roughness of the fabricated crowns. The distribution of all datasets was evaluated for normality using the Shapiro–Wilk test. To test the first null hypothesis (the effect of progressive bur wear) and the second null hypothesis (difference between machines), the manufacturing subgroups and the overall data for the various analyzed crown areas were considered as independent variables, respectively. Due to the unequal sample sizes between the two SM systems, a Welch’s ANOVA followed by Dunnett’s T3 post hoc test was used.

## Results

**Figure 6:**
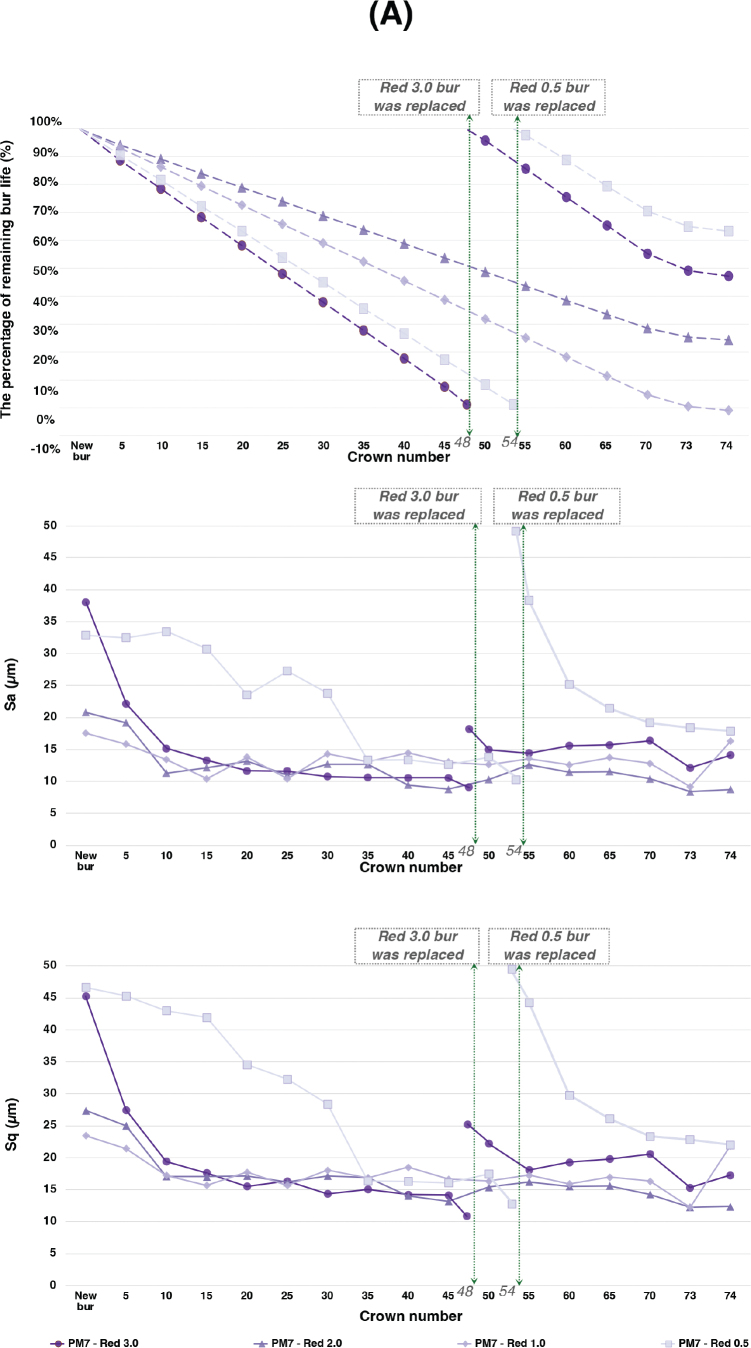

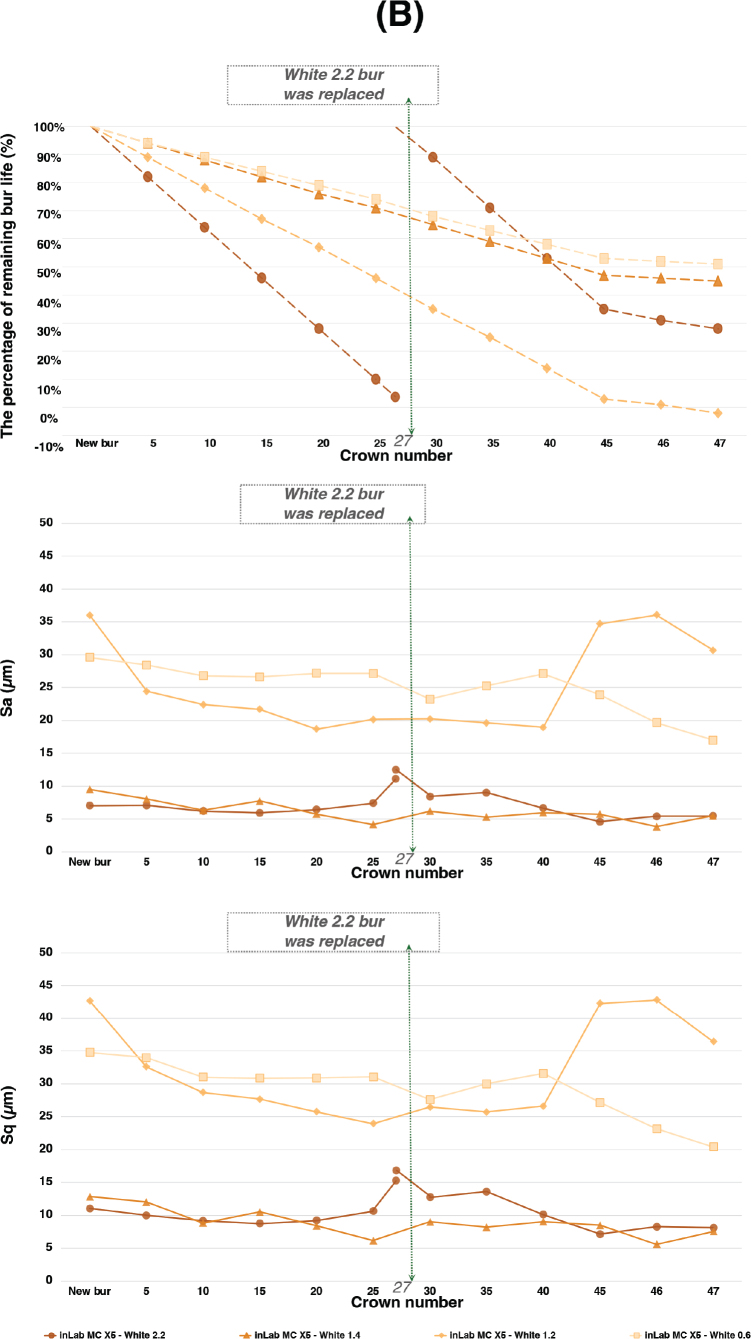
Bur degradation recorded during the manufacturing processes as the remaining bur life in the CAM programs of (A) PM7 diamond burs and (B) inLab MC X5 diamond burs. The changes in surface roughness of the diamond bur tips of (A) the PrograMill PM7 and (B) inLab MC X5 during repetitive milling cycles. Bur degradation recorded during the manufacturing processes as the remaining bur life in the CAM programs of (A) PM7 diamond burs and (B) inLab MC X5 diamond burs. The changes in surface roughness of the diamond bur tips of (A) the PrograMill PM7 and (B) inLab MC X5 during repetitive milling cycles.

The milling experiment continued until the designated reference burs, Red 1.0 mm for PM7; White 1.2 mm for MC X5, completed their manufacturer-specified milling time. During this process, a total of 74 Vita Enamic crowns were milled using the PrograMill PM7, and 47 crowns were milled using the inLab MC X5. Each machine produced an over-milled crown at the end of its respective milling cycle – the 74th crown for PM7 and the 47th for MC X5. Bur replacements were required at different intervals for each machine (Figure 6). The PM7 required replacement of its largest (Red 3.0-mm) and smallest (Red 0.5-mm) burs after milling 48 and 54 crowns, respectively. In contrast, the MC X5 only needed one replacement: its largest bur (White 2.2-mm), after milling 27 crowns. The other burs in the initial sets for both machines completed the entire SM process without needing replacement.

### Milling strategy and bur degradation

Both the PM7 and MC X5 units operated in a ‘high finish/detailed’ milling mode. The total time to mill a single Vita Enamic premolar was similar: 36 min and 30 s on the PM7 and 32 min and 45 s on the MC X5. Although the completion times were comparable, each machine used a different milling strategy, as shown in Table 2. The MC X5 spent approximately half of its overall milling time on bulk material removal, primarily using its largest bur (White 2.2 mm). Fine detailing was performed using two intermediate burs (White, 1.4 mm and 1.2 mm), which, although having similar durations, served different purposes. The White 1.4-mm bur was used to mill the intaglio surface and portions of the outer surface, while the White 1.2-mm bur was dedicated to fine-grinding the remaining external surfaces. Conversely, the PM7 distributed the workload more evenly across its burs, except for the finest bur (Red 0.5-mm). It used a sequential grinding approach, progressing from the largest to the smallest bur for rough-to-fine grinding.

**Table 2:** Milling strategies of the PrograMill PM7 and inLab MC X5 when milling VITA Enamic crowns.

	PrograMill PM7 (Ivoclar Vivadent)				inLab MC X5 (Dentsply Sirona)			
Milling mode	High finish crown with fissure				High detail – normal speed			
Total milling time (per crown)	36 min 30 s				32 min 45 s			
Strategy for each diamond bur (per crown)	Diamond bur	Purpose	Total working time	Percentages of bur life used (%)	Diamond bur	Purpose	Total working time	Percentages of bur life used (%)
	**Red 3.0**	Rough grind out the bulk of the block *(no crown surface is finished by this bur)*	13 min 53 s	2.0 %	**White 2.2**	Rough grind out the bulk of the block *(no crown surface is finished by this bur)*	16 min 49 s	3.6 %
	**Red 2.0**	Shape the internal (fit) surface and outer surface *(but Red 1.0 was used for fine grinding of all areas ground by Red 2.0 bur)*	11 min 37 s	1.0 %	**White 1.4**	Fine grinding of internal (fit) surface, margin and half of the outer surface (close to margin), excluding occlusal surface	6 min 12 s	1.2 %
	**Red 1.0**	Fine grinding of internal and outer surfaces and margin after Red 2.0	8 min 34 s	1.4 %	**White 1.2**	Fine grinding of occlusal surface and the other half of the outer surface (close to occlusal)	7 min 52 s	2.2 %
	**Red 0.5**	Finalize the whole occlusal surface at the end	2 min 26 s	1.8 %	**White 0.6**	Finalize occlusal grooves at the end	1 min 52 s	1.0 %

Regarding the machine-reported lifespan consumption per crown, most of the PM7 burs registered lower percentages of use (Table 2). Notably, the Red 3.0 mm and 1.0 mm of the PM7 consumed over 35% less of their lifespan per crown compared to their MC X5 counterparts. The only exception was the finest bur, where the PM7 showed greater consumption than the MC X5. This corresponded to a difference in milling strategy between the two systems. The PM7 used its 0.5-mm bur to finalize the entire occlusal surface, whereas the MC X5 used its 0.6-mm bur only to define the occlusal grooves.

Surface roughness measurements (Sa and Sq) of diamond bur surfaces from confocal microscopy demonstrated a consistent, gradual decline with progressive use (Figure 6). This reduction is a direct measure of the abrasive surface smoothing as particles are fractured or lost. The initial measurements of post-replacement burs consistently showed roughness levels similar to those of the burs being replaced, indicating consistent quality and uniformity of diamond burs of the same size within each system. Unlike the standardized linear wear assumption reported by CAM software (Figure 6), the PM7 burs displayed a significantly accelerated initial decline in roughness, especially within the first 10 crowns milled. For the smallest bur (Red 0.5-mm), this rapid wear phase extended until around 30 milled crowns (Figure 6A). In contrast, the diamond burs used in the MC X5 unit exhibited a more attenuated rate of roughness decrease. An unusual increase was observed with the 1.4-mm bur, which showed rises in both Sa and Sq during the milling of the last seven crowns (from the 40th to the 47th) (Figure 6B).

Topographical changes were quantified using the Ssk and Sku parameters, which illustrate the evolving distribution of diamond particles with increasing use (Tables 3 and 4). For the PM7 system, Ssk values were highly variable, ranging from approximately −2.5 to +1.0, reflecting significant shifts in surface symmetry. New burs registered values near zero or slightly positive, confirming a peak-dominated surface formed by intact diamond projections. Ssk showed slight negative values during initial use (up to 20 crowns) and became progressively more negative with extended use, indicating surface alteration characterized by peak flattening, valley deepening, and a severely worn texture. High Sku values, often exceeding 10–20, typified the PM7 burs, indicating a sharply peaked, irregular surface with considerable microtopographic instability throughout the wear process. In contrast, MC X5 burs maintained a narrower Ssk range (between −0.4 and +0.8), indicating greater stability in surface asymmetry. Their Sku distribution was also more concentrated, generally ranging from 6 to 14, with fewer extreme spikes, reflecting less critical microtopographic changes. For both systems, the introduction of new replacement burs immediately restored the surfaces to high Sku and slightly positive Ssk values.

**Table 3:** Surface roughness (unit: µm) of diamond burs of the PrograMill PM7.

PrograMill PM7																			
Number of milled crowns	0	5	10	15	20	25	30	35	40	45	48	50	54	55	60	65	70	73	74
**Skewness (Ssk) – asymmetry of the surface height distribution relative to the mean plan** *(negative value means valley-dominated textures and positive values means peak-dominated surfaces)*																			
**Red 3.0**	1.332	0.672	1.135	0.847	1.298	0.875	0.822	0.686	0.476	0.154	−0.367	NR	NR	NR	NR	NR	NR	NR	NR
**Replaced Red 3.0**	0.778	NR	NR	NR	NR	NR	NR	NR	NR	NR	NR	NR	1.064	0.514	0.394	0.542	0.634	−0.970	−0.279
**Red 2.0**	2.886	1.888	1.649	1.623	1.443	1.426	0.818	0.622	0.505	0.333	NR	0.625	NA	0.463	0.887	−0.214	0.208	−0.446	−0.579
**Red 1.0**	0.327	−1.040	−0.907	−1.000	−1.086	−1.023	−1.199	−1.051	−1.284	−1.144	NR	−1.140	NA	−1.123	−1.313	−1.731	−1.649	−2.193	−2.193
**Red 0.5**	1.340	1.472	1.438	1.142	1.248	1.107	0.931	−1.628	−1.664	−2.083	NR	−1.947	−2.549	NR	NR	NR	NR	NR	NR
**Replaced Red 0.5**	1.214	NR	NR	NR	NR	NR	NR	NR	NR	NR	NR	NR	NR	1.241	1.232	1.123	1.021	0.903	−0.152
**Kurtosis (Sku) – sharpness of the surface profile** *(value above 3 means a spiked topography and value below 3 means flatter features)*																			
**Red 3.0**	6.397	5.700	7.382	10.861	8.305	12.986	9.778	6.28	3.549	3.219	2.488	NR	NR	NR	NR	NR	NR	NR	NR
**Replaced Red 3.0**	9.812	NR	NR	NR	NR	NR	NR	NR	NR	NR	NR	NR	14.666	6.175	5.293	4.774	3.160	4.164	3.055
**Red 2.0**	29.860	24.582	21.542	19.751	13.386	10.441	7.113	8.722	6.389	6.932	NR	6.680	NA	5.710	4.628	5.917	4.600	4.982	4.999
**Red 1.0**	16.530	15.367	7.909	6.032	6.913	7.462	4.646	4.543	4.204	4.495	NR	4.084	NA	4.042	4.068	3.879	3.493	3.377	3.833
**Red 0.5**	27.931	23.349	11.296	15.996	9.132	3.135	2.976	3.035	2.708	2.580	NR	2.274	2.258	NR	NR	NR	NR	NR	NR
**Replaced Red 0.5**	28.070	NR	NR	NR	NR	NR	NR	NR	NR	NR	NR	NR	NR	25.561	12.449	12.457	5.297	2.198	2.140

NR indicates no results are available because the diamond bur was either not used, replaced, or measurement was not taken.

**Table 4:** Surface roughness (unit: µm) of diamond burs of the inLab MC X5.

inLab MC X5													
Number of milled crowns	0	5	10	15	20	25	27	30	35	40	45	46	47
**Skewness (Ssk) – asymmetry of the surface height distribution relative to the mean plan** *(negative value means valley-dominated textures and positive values means peak-dominated surfaces)*													
**White 2.2**	1.270	0.644	0.439	0.413	−0.393	+0.153	−1.151	NR	NR	NR	NR	NR	NR
**Replaced White 2.2**	1.802	NR	NR	NR	NR	NR	NR	1.186	1.171	0.502	0.606	0.227	−0.599
**White 1.4**	1.474	1.246	0.875	0.545	−0.014	−0.056	NR	−0.089	−0.258	−0.425	−1.509	−1.615	−1.923
**White 1.2**	0.979	0.418	0.432	0.259	0.170	−0.003	NR	0.090	−0.025	−0.060	−0.199	−0.112	−0.446
**White 0.6**	1.099	0.974	0.774	0.694	0.627	0.697	NR	0.698	0.587	0.487	0.342	0.441	0.454
**Kurtosis (Sku) – sharpness of the surface profile** *(value above 3 means a spiked topography and value below 3 means flatter features)*													
**White 2.2**	18.740	10.972	6.453	7.087	6.746	6.982	4.339	NR	NR	NR	NR	NR	NR
**Replaced White 2.2**	11.134	NR	NR	NR	NR	NR	NR	11.633	10.507	9.007	8.956	7.748	4.043
**White 1.4**	14.171	13.214	10.268	10.804	9.589	9.246	NR	8.353	8.713	8.958	6.487	6.785	6.938
**White 1.2**	4.861	4.294	4.316	3.973	3.695	3.349	NR	3.621	2.885	2.676	2.260	2.231	2.281
**White 0.6**	2.354	2.376	2.283	2.245	2.265	2.021	NR	2.132	1.911	1.991	1.933	1.910	1.917

NR indicates no results are available because the diamond bur was either not used, replaced, or measurement was not taken.

**Figure 7:**
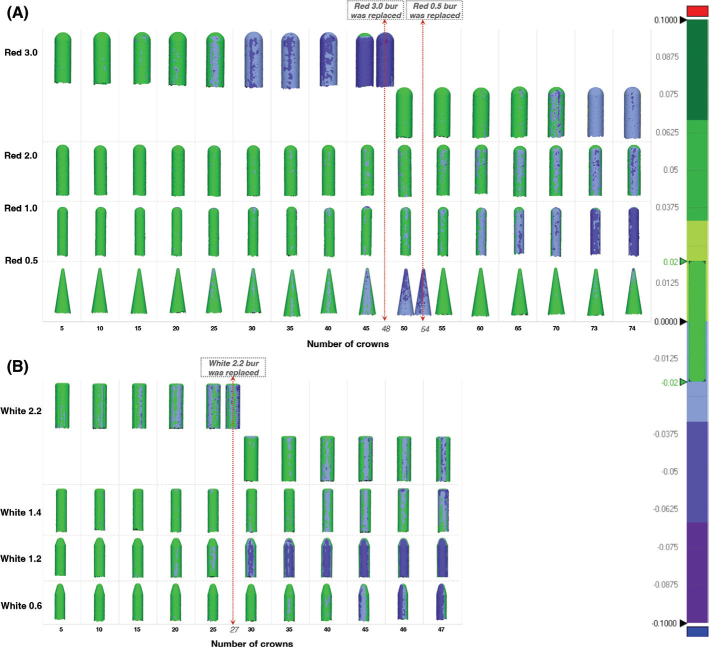
Three-dimensional color deviation maps of diamond burs used in the (A) PrograMill PM7 and (B) inLab MC X5 across sequential milling intervals.

The 3D color deviation maps in Figure 7 clearly reveal the spatial distribution and development of bur wear. Negative deviations in surface height (purple/blue areas) consistently appear along the leading edges and diamond-coated flutes, corresponding to the primary contact zones during ceramic cutting. These regions indicate material loss due to diamond particle detachment and bonding matrix erosion. While abrasive depletion occurred between the 20th and 30th crowns for both systems, the MC X5 demonstrated an earlier and more extensive expansion of smoothing or wear areas (i.e. beyond the 30th to 40th crowns). Since the largest PM7 bur (Red 3.0-mm) was employed for a longer duration (i.e. machining more crowns) than the MC X5 counterpart (White 2.2-mm bur), the PM7 bur ultimately exhibited a more severe, dark-purple wear zone.

**Figure 8:**
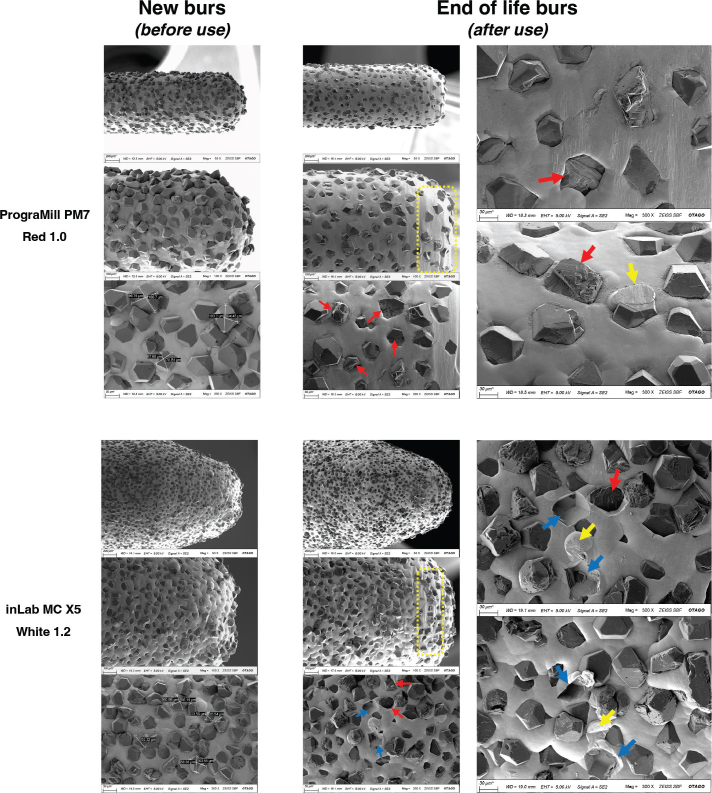
Scanning electron microscopy (SEM) micrographs of diamond burs before and after use (PrograMill PM7 after milling 74 crowns and inLab MC X5 after milling 47 crowns). Red arrows indicate the chipped or broken diamond particles; Blue arrows indicate the diamond particle loss; Yellow arrows and rectangles denote the wear pattern on the matrix.

SEM micrographs provided morphological evidence of surface degradation in the reference burs (Red 1.0 for PM7 and White 1.2 for MC X5) both before use and after their lifespan (Figure 8). The new PM7 bur possessed larger (nearly 100 µm diameter) and more regularly-shaped, angular diamond particles with minimal exposure of the matrix. The unused MC X5 bur, however, already showed minor chipping at the edges of some particles. After milling, both worn surfaces displayed degradation features: crystal edge rounding, facet chipping, and micro-cavity formation, confirming a transition to a smoother, depleted morphology. The specific wear mechanisms differed between the two systems. The PM7 worn bur primarily exhibited brittle fracture, evidenced by micro-cracking, edge rounding, facet spalling, and some particles appearing partially sheared off. The exposed matrix displayed homogeneous grinding paths. In contrast, the MC X5 worn bur exhibited hexagonal or irregular cavities within the bonding matrix, indicating that complete particle detachment (pull-out) was the dominant mechanism. The matrix surface was uneven, with prominent sections that were either pulled away or severely flattened.

**Figure 9:**
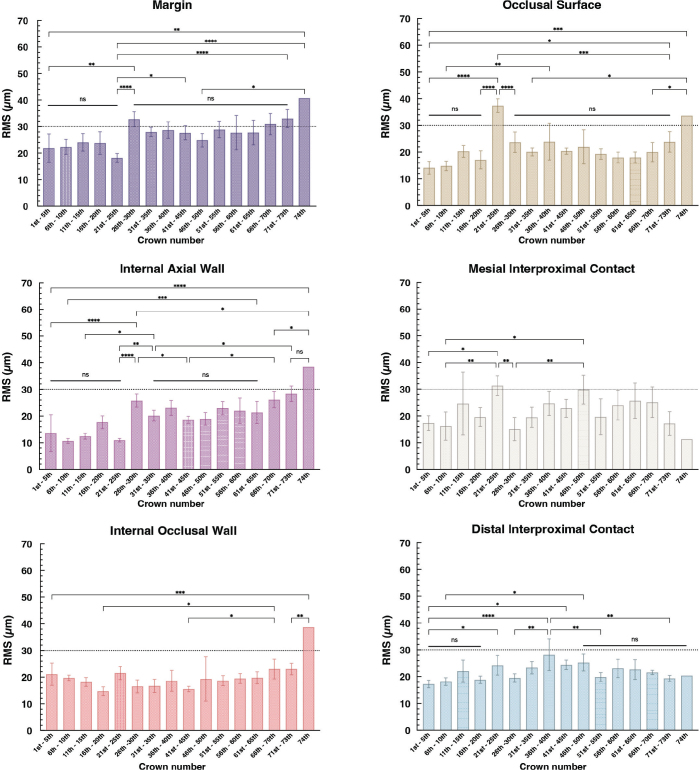
The average root mean square (RMS) ± standard deviation (SD) (unit: µm) of various anatomical regions of PICN crowns manufactured from the PrograMill PM7.

### Trueness of PICN crowns

The overall trueness of the manufactured PICN crowns is summarized in Table 5, where root-mean-square (RMS) values indicate trueness and SDs represent precision. Comparative analysis revealed that the PM7 system achieved significantly superior trueness with lower RMS values for the intaglio regions, whereas the MC X5 demonstrated significantly higher trueness when shaping the outer crown surfaces (*p* < 0.0001). No statistically significant difference was found between the two systems regarding margin and internal axial wall machining (*p* = 0.9979 and *p* = 0.0791, respectively), despite the MC X5 displaying slightly lower RMS values. In terms of precision, the MC X5 produced lower SDs across most anatomical regions, except for the mesial interproximal contact, indicating a more precise SM process. Furthermore, trueness varied significantly across some anatomical regions within the same system. For instance, the marginal surfaces milled by the PM7 exhibited significantly higher RMS values (i.e. lower trueness) than other areas (*p* < 0.002), whereas in the MC X5, the internal occlusal surface showed significantly higher RMS values than other crown regions (*p* < 0.0001).

**Table 5:** The mean ± standard deviation (SD) (unit: µm) of root mean square values of crowns manufactured by the PrograMill PM7 and inLab MC X5 across different analyzed regions.

Crown region of analysis	PrograMill PM7	inLab MC X5
Overall inner surface	22.21 ± 4.31^A,B^	28.10 ± 2.93^C^
Margin	26.73 ± 5.35^C,D^	25.20 ± 3.54^D,E^
Internal axial wall	19.53 ± 6.30^B,H^	22.68 ± 3.57^B,E^
Internal occlusal wall	19.23 ± 4.36^H,I^	37.77 ± 4.09^G^
Overall outer surface	24.80 ± 4.90^A,D,E^	17.89 ± 2.76^H^
Occlusal surface	20.96 ± 6.27^B,I^	13.70 ± 1.65^J^
Mesial interproximal contact	22.16 ± 2.93^B,E,I^	14.48 ± 3.29^J,K^
Distal interproximal contact	21.89 ± 3.87^B^	17.24 ± 4.34^H^

Means followed by the same superscript letter are not significantly different (*p* > 0.05) according to Dunnett’s T3 multiple comparisons test. The analysis compares all mean values simultaneously.

**Figure 10:**
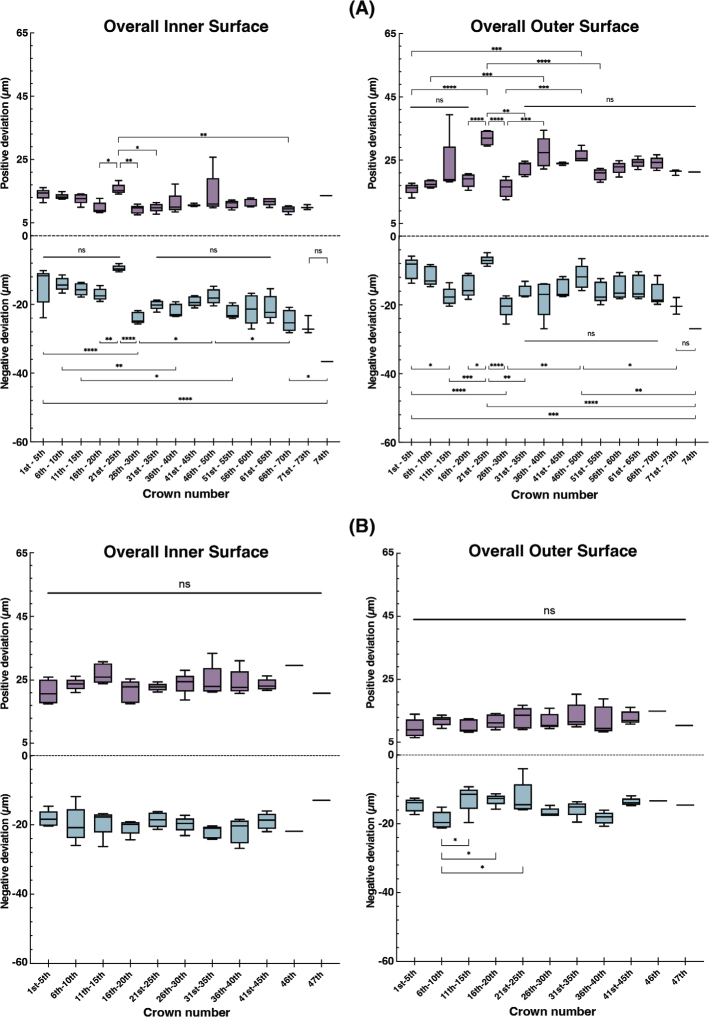
The average root mean square (RMS) ± standard deviation (SD) (unit: µm) of various anatomical regions of PICN crowns manufactured from the inLab MC X5.

The impact of continuous milling on crown trueness is shown in Figures 9 and 10, which display the average RMS values for different anatomical surfaces produced by the PM7 and MC X5 systems, respectively. Overall, the PM7 unit demonstrated a more pronounced sensitivity to cumulative milling cycles. For the crown internal surfaces machined by the PM7, a progressive increase in RMS values (indicating a gradual loss of trueness) was observed. While no significant differences appeared during the first 25 crowns (*p* > 0.05), the average RMS values for the margin and internal axial wall rose significantly from the 26th–30th crowns onward, differing markedly from the initial five crowns (*p* = 0.0019 and *p* < 0.0001, respectively). Although the inner surfaces generally maintained RMS values below 30 µm, the final crown (74th), produced by an overused bur, showed a sharp and notable rise to nearly 40 µm (*p* < 0.05). The external surfaces milled by the PM7 did not follow a linear degradation trend but rather exhibited staged fluctuations, especially at the interproximal contacts. The RMS values increased markedly between the 1st–5th and 21st–25th subgroups (*p* < 0.0251), then declined during the 26th–30th interval, with this pattern repeating in the 51st–55th subgroup. In contrast, the MC X5 system demonstrated greater stability, as repeated milling cycles did not significantly affect the RMS values of the marginal and occlusal surfaces (*p* > 0.05). Although the final (47th) crown did not exhibit pronounced global degradation, the penultimate crown (46th) demonstrated a distinct spike in RMS values for the internal axial wall and mesial contact compared to the first crown (*p* = 0.0029, *p* = 0.0006). Across all anatomical regions, the internal occlusal wall machined by the MC X5 consistently displayed the highest RMS values, with all measurements exceeding 30 µm regardless of bur deterioration.

**Figure 11:**
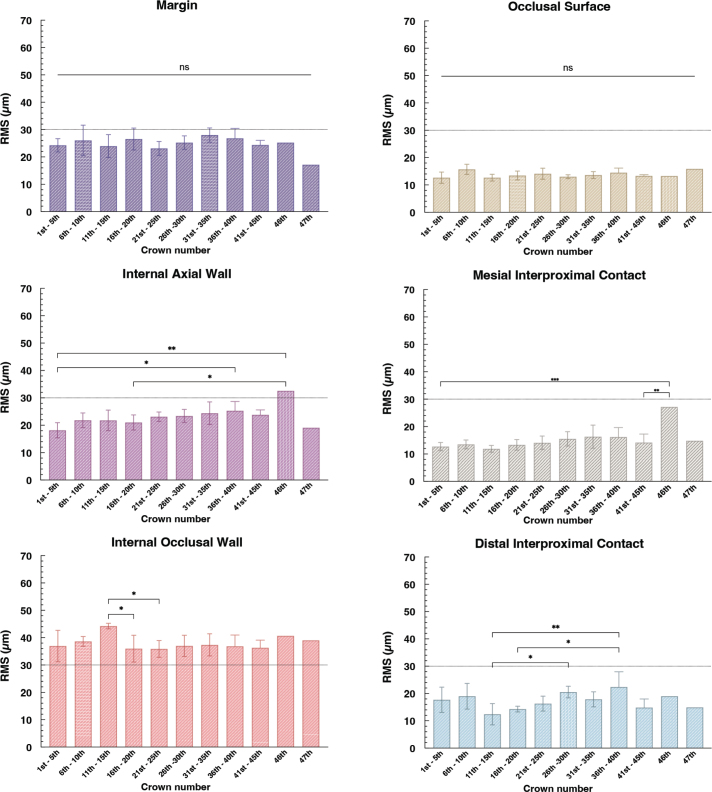
The average positive and negative deviations (unit: µm) of the overall inner and outer surfaces machined by (A) the PrograMill PM7 and (B) the inLab MC X5 systems.

The progression of average positive (under-milling) and negative (over-milling) deviations for the overall inner and outer surfaces is illustrated in Figure 11. For the PM7 system, the positive deviations of the inner surfaces remained relatively stable, with a solitary transient spike occurring in the 21st–25th subgroup, which showed notable differences with its previous and subsequent subgroups (*p* = 0.0149, *p* = 0.0060, respectively) (Figure 11A). However, a distinct trend was observed in the negative deviations, which increased gradually for both inner and outer surfaces, culminating in distinct trends between the first and last crowns (*p* < 0.0001, *p* = 0.0005). The positive deviations of the outer surfaces produced by the PM7 showed a progressive increase overlaid with cycle-to-cycle fluctuations, although the first 20 crowns and the last 10 subgroups (from 31st to 74th crowns) remained statistically comparable (*p* > 0.05). In comparison, the MC X5 demonstrated more consistent deviation patterns, with notable differences occurring primarily during mid-sequence intervals (comparing the 6th–10th to 11th–25th, 16th–20th, and 21st–25th; *p* = 0.0216, *p* = 0.0388, *p* = 0.0230, respectively) (Figure 11B). This suggested that while the MC X5 maintains global stability, subtle shifts in machining accuracy occur during the mid-life phase of the diamond burs.

**Figure 12:**
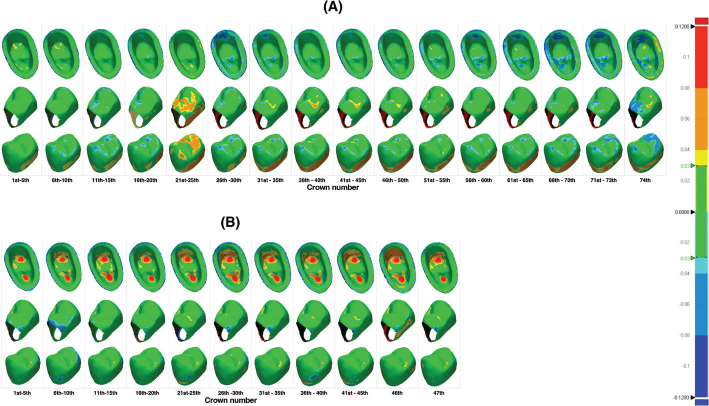
Sequential 3D color deviation maps (unit: mm) of PICN crowns milled with (A) the PrograMill PM7 and (B) the inLab MC X5.

The 3D color deviation maps provide critical insight into the local distribution of errors, revealing distinct discrepancy patterns between the two systems (Figure 12). Red/orange zones indicate positive deviations (under-milling), while blue zones indicate negative deviations (over-milling). The PM7 exhibited a tendency toward over-milling as the SM process progressed. Initially, slight yellowing (minor under-milling) on the internal occlusal walls diminished to a ‘safe’ green zone by the 16th–20th crowns. Subsequent cycles produced progressively enlarging blue zones around the intaglio occlusal fossa and internal buccal axial wall, indicating severe over-milling (ranging from 30 µm to −80 µm). On the external surfaces, orange/yellow regions (deviations < 80 µm) emerged on the occlusal fissures and buccal/distal surfaces prior to the replacement of the 0.5-mm bur (54th crown). Following this, both blue and orange zones expanded, resulting in a catastrophic mixed pattern of undercuts and overcuts on the 74th crown, consistent with the numerical results in Figure 11A. The MC X5 system, on the other hand, displayed a systematic tendency toward under-milling, particularly in critical fit areas. While the external occlusal surfaces were superiorly accurate with only minor yellowing in fissures, the internal cusp tips appeared red/orange from the very first subgroup, indicating substantial positive deviations over 80 µm (approaching 120 µm) and representing a systematic limitation of the 1.4-mm bur diameter rather than a loss of milling accuracy due to the bur degradation. However, this specific localized inaccuracy was masked in the global average RMS values (Figure 10) but may induce a potential clinical fit issue. This phenomenon is categorized as a system-inherent geometric limitation rather than a wear-dependent error. As milling cycles were repeated, these undermilled zones spread to the internal axial walls, reaching deviations of approximately 80 µm.

### Surface roughness of PICN crowns

**Figure 13:**
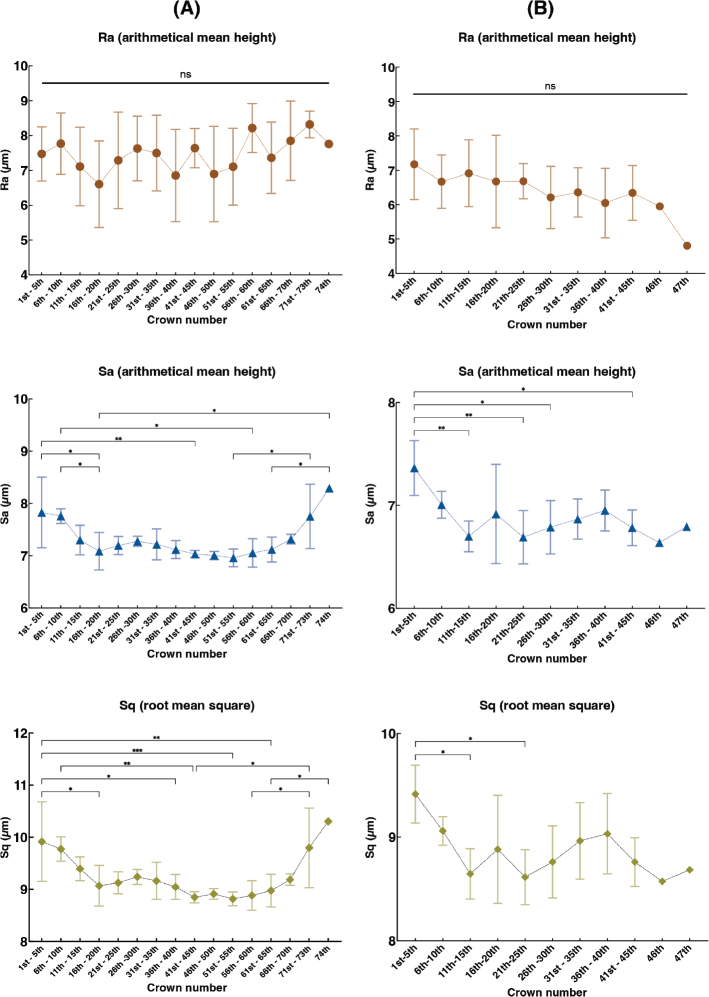
Average surface roughness (Ra, Sa, Sq) ± standard deviation (SD) (unit: µm) of PICN crowns manufactured from (A) the PrograMill PM7 and (B) the inLab MC X5.

The variations in surface roughness parameters (Ra, Sa, Sq) for PICN crowns across successive milling cycles are depicted in Figure 13. The profile roughness (Ra) of the PM7 group exhibited a stochastic pattern characterized by marked fluctuations and distinct peaks during the mid-to-late cycles, yet the overall trend indicated a gradual, cumulative increase in micro-texture roughening (Figure 13A). However, the areal roughness parameters (Sa and Sq) provided a more comprehensive insight, revealing a distinct three-stage behavior. The initial phase, the first 20 crowns, demonstrated a rapid improvement in surface quality, with roughness dropping by approximately 1 µm. This was followed by a long, stable plateau characterized by a polishing effect until the 55th crown, which exhibited the lowest recorded roughness values. Significant differences were observed between it and the initial-stage (1st–5th) crowns (*p* = 0.0013 for Sa; *p* = 0.0003 for Sq). From the 56th crown onward, surface quality degraded rapidly, marked by a pronounced, steep increase in both Sa and Sq, with significant differences compared to the 74th crown (*p* = 0.0195 and *p* = 0.0209, respectively). Moreover, variability was non-uniform, with the initiation (1st–5th) and termination (71st–73rd) phases of the milling sequence exhibiting significantly higher SDs in comparison to the stable mid-stage. As expected, due to the sensitivity of RMS parameters to extreme outliers like deep valleys or high peaks, Sq values consistently exceeded Sa by about 2 µm throughout the entire process timeline.

The crowns manufactured by the MC X5 system showed a significantly different pattern in surface roughness (Figure 13B). Profile roughness (Ra) showed a gradual, linear downward trend from 7.17 µm to 4.80 µm, with no statistically significant differences across subgroups. Despite these, the areal parameters (Sa and Sq) revealed a more complex, non-linear behavior. A significant linear decline occurred during the first 15 crowns (*p* = 0.0084 for Sa; *p* = 0.0239 for Sq), suggesting an initial conditioning of the burs. This trend, however, was interrupted by a sudden spike in roughness within the 16th–20th subgroup. The crown roughness resumed a progressive upward trend from the 21st–25th crowns and attained a peak value of 6.95 µm in Sa and 9.03 µm in Sq during the 36th–40th subgroup, before declining once more in the final stage. In contrast to the PM7 group, the MC X5 group exhibited marginally larger SDs within corresponding subgroups for Sq, indicating a lower degree of predictability in the microtopography of the milled restoration surfaces.

## Discussion

This study primarily aimed to quantify diamond bur deterioration during repeated milling cycles and to correlate this wear with the dimensional trueness and surface roughness of PICN restorations. Based on the results, both null hypotheses, that no significant differences would exist between milling systems and that progressive bur wear would not affect restoration quality, were rejected. The primary finding of this investigation was that the PrograMill PM7 showed a significantly lower rate of bur degradation per machined crown compared to the inLab MC X5 system. Differences in trueness were also observed: the PM7 produced more accurate intaglio (inner) surfaces (22.21 ± 4.31 µm), while the MC X5 achieved higher external surface trueness (17.89 ± 2.76 µm). Furthermore, while bur degradation was confirmed as a determinant factor in the surface roughness of PICN crowns, the nature and direction of this roughness change varied notably between the two SM systems. The MC X5 displayed a linear decrease in roughness, whereas the PM7 showed a non-linear, U-shaped pattern – with an initial reduction followed by an increase. These findings suggest that the distinct tool path strategies and bur microarchitectures (e.g. diamond grit binder interactions) of each system play a critical role in degradation kinetics, which in turn, determine the final quality of PICN dental restorations. From a practical cost-benefit perspective, this study’s findings also suggest that laboratories should consider replacing diamond burs before the CAM software’s maximum recommended lifespan when manufacturing PICN restorations. A preemptive bur replacement protocol is highly advisable to guarantee consistent restorative quality.

**Figure 14:**
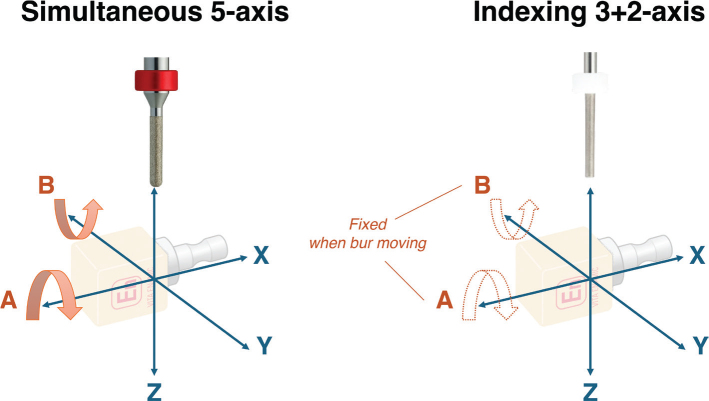
Comparison between the simultaneous 5-axis and indexing 3 + 2-axis machining kinematics.

Variation in preset milling strategies across dental CAM programs arises because there is no single, universal mathematical standard for subtractive material removal. Each software vendor develops proprietary algorithms that balance competing optimization parameters, such as surface quality, machining time, and tool longevity, resulting in distinct G-code outputs for identical digital designs [32, 33]. These CAM solvers (calculation engines) determine the specific toolpath strategies (e.g. raster, spiral, or offset milling), but the particular kinematics and dynamics of the milling hardware fundamentally constrain their calculations [34, 35]. Both systems used in this study are classified as 5-axis SM machines. In standard dental CNC setups, the three translational linear axes (X, Y, and Z) control the spatial position of the spindle (cutting tools) relative to the workpiece, while the two rotary axes, typically the A-axis (rotation around X) and B-axis (rotation around Y), control the angular orientation of the block/blank [34, 36]. This setup offers a clear advantage over fewer-axis models by allowing access to deep undercuts or complex shapes and ensuring that the cutting tool can be optimally oriented relative to the workpiece [36]. This capability is essential for reducing scallop height and maintaining ideal chip loads, which refers to the thickness of material removed per cutting edge (flute) per revolution [37]. Maintaining an ideal chip load is crucial; otherwise, low loads cause friction and premature tool wear, or high loads may result in tool fracture and poor surface quality [38, 39]. Although both machines share this geometric feature, they operate on fundamentally different kinematic control philosophies, which directly influence the complexity of the toolpath and the trade-off between machining speed and mechanical rigidity [36, 40]. The PrograMill PM7 utilizes simultaneous 5-axis kinematics, where the rotary and linear axes undergo continuous, synchronized interpolation (Figure 14). This capability allows the machine to maintain an optimal tool-to-surface angle (often perpendicular to the surface normal) throughout complex curvatures, effectively managing undercuts and ensuring consistent chip load without stopping to reposition [36, 41]. In contrast, the inLab MC X5 operates primarily as a 3 + 2 or indexing 5-axis system. In this configuration, the rotary axes (A and B) orient and lock the workpieces into a fixed position, after which the three linear axes operate the burs to perform the actual material removal. While simultaneous milling offers superior agility for complex geometries, 3 + 2 machining often provides greater mechanical rigidity and stability during the cutting pass, potentially reducing vibration [42, 43].

The distinct kinematic strategies employed by the two SM systems fundamentally govern the observed disparities in diamond bur wear and progression patterns. By utilizing a positional (3 + 2) indexing configuration, the MC X5 relies primarily on linear-axis movements with a static tool orientation, a constraint that restricts the cutting interface to specific zones of the diamond particles [44]. This localized, repetitive, unidirectional stress would lead to the development of asymmetric ‘wear stripes’ or flat spots, rather than uniform abrasion. The resulting anisotropic contact likely causes increased radial cutting forces and frictional heat in localized bands [45], a mechanism directly associated with the striated purple wear patterns observed in the deviation maps and the extensive pull-out of diamond particles (adhesive failure identified in the SEM analysis). In contrast, the simultaneous 5-axis interpolation of the PM7 continuously modulates the tool-to-workpiece angle along the toolpath. This dynamic distribution of abrasive load across a broader, circumferential surface area mitigates the formation of deep wear bands, resulting in significantly more homogeneous surface degradation. Such uniform stress distribution also appears to alter the dominant mode of failure. Rather than the complete particle detachment observed in the MC X5, the PM7 burs exhibited abrasive wear and microchipping, with diamond fragments remaining mechanically interlocked within the binder matrix. Independent of these kinematic factors, the intrinsic morphology of the cutting tool played a defining role in longevity. The diamond particles on the PM7 burs were approximately 50% larger than those on the MC X5 counterparts, providing a greater volume of abrasive material to resist wear. This combination of increased particle mass and distributed wear patterns effectively extends the functional lifespan of cutting tools [46], distinguishing the capacity of PM7 to manufacture a significantly higher volume of restorations per bur set.

Beyond their influence on bur degradation, the choice of kinematic strategy fundamentally dictates the physical accessibility of the cutting tool to complex geometries, thereby directly governing machining accuracy [47, 48]. The simultaneous 5-axis motion of the PM7 enables continuous tool reorientation, maintaining optimal cutting angles and providing access to complex geometries. However, this dynamic tilting has two notable consequences that affect both the tools and the restorations. As it distributes the cutting load across a greater surface area of the cutting edges of burs (flutes), this prevents localized stress and friction that typically cause rapid failure [49]. The resulting more uniform and even wear pattern could therefore explain the increased available working time that the manufacturer suggests for their burs. On the other hand, this continuous reorientation can inadvertently lead to aggressive material removal in concave regions, resulting in widespread over-milled surfaces (blue zones) observed in the deviation maps. Such excessive subtraction results in thinning of the restoration and enlarged cement spaces, defects that compromise fracture resistance, retention and occlusal load distribution [10]. Interestingly, a transient increase in positive deviations (i.e. the appearance of an orange/yellow region) was observed between the 21st and 55th crowns, followed by a recovery of trueness. This fluctuation is likely attributable to the specific wear cycle of the 0.5-mm finishing bur, which was tasked with finalizing the entire occlusal surface. Its progressive dulling caused temporary under-cutting until its replacement after the 54th crown, restoring cutting efficiency. On the other hand, the indexed 3 + 2 kinematics of the inLab MC X5 demonstrated limited accessibility to steep or constrained anatomies, particularly within the intaglio surface [50]. While this strategy produced superior trueness on the less complex outer crown surfaces, it generated significant residual uncut material (under-milling) internally. This positive deviation creates internal interferences that impede the complete seating of the restoration. From a clinical perspective, such interferences necessitate extensive chairside adjustments; if left uncorrected, they result in elevated marginal gaps. Although luting cements can initially fill these discrepancies, the resultant thick cement layer is prone to dissolution, potentially exposing the gap to plaque accumulation, increasing the risk of secondary caries, and periodontal inflammation [51]. As a consequence, both error modalities, that is, over-milling and under-milling, pose distinct but significant threats to the functional longevity, mechanical performance and biological integrity of the final restoration.

The baseline surface quality of the PICN crowns is fundamentally dictated by the system’s CAM strategy, specifically the stepover (the lateral distance between the adjacent milling paths) and the resulting scallop height (the microscopic ridges of residual material left between these passes). However, the progressive degradation of the diamond burs significantly compromised the manufacturing accuracy and cutting efficiency. This alters the effective chip load (the physical volume of material removed by the bur during a single rotation), leading to increased friction and erratic material removal. The specific manifestation of these dimensional errors was fundamentally dictated by the interaction between the physical state of the burs and the motion architecture of the respective CAM system. Discrepancies were observed across crown regions within the PM7 group. External surfaces displayed increased positive deviations (under-milling) due to severe smoothing of the 0.5-mm finishing bur, resulting in a significant decrease in cutting efficiency; meanwhile, internal surfaces exhibited progressively increasing negative deviations (over-milling). This divergence is best understood through the biomechanics of cutting. As diamond abrasive particles undergo attrition and microfracture, their sharp edges become blunted, forcing a tribological shift from efficient material shearing to insufficient ploughing and rubbing [52]. Within the concave internal regions, the mechanics of simultaneous 5-axis interpolation appear to interact distinctively with the increased regular forces of a worn bur [53]. The intensified rubbing action, combined with the dynamic multi-axis approach, facilitated deeper penetration into the cavity, resulting in the observed cumulative over-milling. However, the MC X5 displayed a more uniform trend of increasing under-milling across both inner and outer geometries. This pattern suggests that as the bur dulls, the conservative 3 + 2 indexing lacks the kinematic agility to force the worn bur into steep or constrained topography, resulting in insufficient material removal. These system-specific responses extend the understanding of the findings of Azarbal et al. [54], who similarly observed a gradual, though system-dependent, drift in marginal integrity across sequential milling cycles of PICN copings.

With regard to clinical acceptability, the consensus on ideal trueness in SM is a deviation below 30 µm, with a maximum tolerance threshold generally recognized at 120 µm [30, 31]. Based on the RMS results reported in this study, the PM7 system maintained the trueness within the ideal < 30 µm range across most anatomical regions, a performance corroborated by color maps showing overall deviations mostly within ±80 µm. The substantial decrease in surface trueness associated with the overused bur (74th crown) warrants caution, as this trend may exceed acceptable limits and potentially compromise precision in high-volume manufacturing. The MC X5 system presented a more complex scenario where average metrics masked localized failures. Although the average RMS for the internal occlusal areas was a moderate approximately 40 µm, the 3D deviation maps revealed critical localized inaccuracies. Internal cusp tip regions appeared as dark red zones, indicating positive deviations exceeding 80 µm and approaching the 120 µm unacceptable limit. This specific error is attributable to limitations in tool geometry, as the 1.4-mm cylindrical bur possesses a flat end tip and a relatively large diameter and is unable to fully access or replicate internal occlusal morphologies. Distinguishing these baseline geometric errors from the progressive degradation-induced errors seen in other regions is crucial for a correct interpretation of the milling accuracy of the MC X5 unit. When combined with the static limitation of the indexed 3 + 2 orientation, it physically restricts access to acute internal line angles, resulting in substantial material removal insufficiency regardless of the bur wear state [55]. Clinically, this implies that while bur degradation affects overall surface quality, the internal trueness in deep occlusal areas is pre-determined by the chosen tool geometry of the CAM system.

The surface roughness of the PICN crown margins, ranging from 7 µm to 10 µm, reflects the complex interaction between the material’s hybrid microstructure and the wear conditions of SM. This level is significantly higher than values usually reported for brittle glass-ceramics (generally below 3 µm) [26, 56, 57], likely due to the different machining responses of the dual-network structure. While the ceramic phase chips away, the polymer network deforms elastically and recovers, creating noticeable topographic relief at the interface [2, 58]. Furthermore, the reliance on bar- or disc-shaped specimens in most previous studies limits the clinical translatability of their findings. In contrast to the uniform, linear toolpaths utilized for flat surfaces, the SM of complex anatomical features necessitates speed changes and multi-axis reorientation. These dynamic kinematic conditions result in fluctuating mechanical loads and localized stress concentrations on the burs, which are not observed during the machining of simpler geometries. In SM, marginal surfaces are primarily machined by the tip of the rotary bur. The circular cutting motion at the bur tips creates more pronounced scalloping, microchipping, and material plucking, which are reliably captured by confocal microscopy [59, 60]. As bur degradation progresses, the worsening of these topographical irregularities carries severe clinical implications that directly threaten the longevity of the restoration. These topographical irregularities have important clinical implications. Deep valleys and sharp peaks act as stress concentrations, increasing the risk of microcrack initiation under occlusal load [61] and disrupting the hydrodynamics of cement flow, potentially leading to irregular film thickness and reduced retention [62]. From a biological perspective, this surface texture provides a niche for accelerated biofilm accumulation, underscoring the necessity for post-milling polishing [63]. This demonstrates that PICN crowns made at the end of bur set lifespan have a considerably higher initial risk of clinical failure. Clinicians and technicians should understand that restorations created with worn burs may require much more detailed and time-consuming post-milling polishing procedures to achieve long-term clinical success.

The progression of this roughness throughout the bur lifespan was fundamentally dictated by the specific motion strategy used. As diamond particles dulled, their cutting efficiency decreased, and their interaction with the PICN material shifted from shearing to frictional ploughing [64]. This ‘glazing effect’ can temporarily smooth the marginal surface, causing a gradual reduction in roughness. However, the later-stage behavior depends on machine kinematics. In systems such as the MC X5, this burnishing trend persists, whereas in simultaneous 5-axis systems such as the PM7, the dynamics of worn burs and machined surfaces may tear the polymer matrix, reintroducing scalloping or over-milling, resulting in a late-stage roughness rebound [65]. Allegedly, the PM7 system showed a characteristic U-shaped trajectory, while the MC X5 showed a steady decrease in roughness. Another contributing factor may be related to the different total numbers of crowns milled in the two systems. The PM7 showed its typical late-stage increase in surface roughness only after about the 51st crown, while the MC X5 workflow finished at 47 crowns, indicating a divergence in trends due to the difference in cumulative bur usage. Among the few studies that have examined the effect of bur wear on the surface roughness of ceramic specimens, Ahmed and Owen [66] reported a 21% decrease with the MC X5 unit. However, Simba et al. [67] argued the opposite, reporting a slight increase in Ra for machined lithium disilicate specimens. These findings highlight the potential influence of material type, underscoring the need for continued investigation in this field.

The trueness and surface roughness of PICN restorations produced through SM are critical determinants of long-term clinical success and biological integration. While this study demonstrated that these surface attributes are affected by bur deterioration and the specific milling unit used, it has certain limitations. Although two distinct milling strategies were assessed, isolating kinematic parameters such as bur geometry, spindle speed, and feed rate, was not achievable, as these variables are proprietary presets defined by the manufacturers. The differences observed between the current findings and previous literature further indicate that the quality of the restorations is highly-dependent on the specific material composition. Future investigations are required to establish comparable wear-quality correlations across other CAD/CAM restorative classes. Another limitation of the present *in vitro* study is the inability to quantify real-time temperature fluctuations at the bur-material interface during the SM processes. Consequently, future research should aim to clarify how these parameters affect the longevity of burs and the surface quality of the restorations produced. Furthermore, the sequential fabrication of crowns introduces a data dependency related to cumulative bur wear. Although crowns were grouped into wear phases, analyzing them as independent observations, rather than using a repeated-measure framework, introduces a risk of pseudoreplication. Lastly, another limitation is the absence of physical seating validation. While localized digital deviations approached clinical thresholds, these findings must be interpreted with caution regarding their absolute impact on the final seated restoration adaptation.

## Conclusion

Within the limitations of this *in vitro* study, the following conclusions can be drawn:

- The PrograMill PM7 demonstrated a longer machine-reported bur lifespan (74 vs. 47 crowns for the designated reference bur). Although progressive wear leads to trueness decline in both systems, the PrograMill PM7 system tends toward over-milling (negative deviation), especially after the 25th crown. Conversely, the inLab MC X5 mainly causes under-milling (positive deviation).
- Bur degradation mechanisms are system-specific, that is, while the inLab MC X5 system is characterized by complete dislodgement of diamond particles, chipped and worn edges are common failure modes for PrograMill PM7 burs.
- For subtractively manufactured PICN crowns, the PrograMill PM7 yields superior intaglio surface trueness, especially on the internal occlusal surfaces, whereas the inLab MC X5 achieves significantly higher trueness on the external crown surfaces.
- The degradation of inLab MC X5 burs results in a gradual decrease in the surface roughness of fabricated crowns. On the other hand, the PrograMill PM7 initially shows a decline in roughness over the first 55 crowns, followed by an accelerated increase afterward.
- Bur replacement strategies should consider not only manufacturer-reported lifespan but also the specific error modes characteristic of each system. Rather than waiting for machine-prompted tool failure, laboratories should consider pre-emptive replacement strategies tailored to the onset of these progressive degradation patterns to ensure consistent restorative quality.
